# Livestock Blastoids: In Vitro Blastocyst Models for Embryogenesis and Reproductive Technologies

**DOI:** 10.1111/rda.70262

**Published:** 2026-09-30

**Authors:** Cristina A. Martinez‐Serrano, Jun Wu

**Affiliations:** ^1^ Department of Molecular Biology University of Texas Southwestern Medical Center Dallas Texas USA; ^2^ Department of Biotechnology National Institute for Agriculture and Food Research and Technology (INIA‐CSIC) Madrid Spain; ^3^ Hamon Center for Regenerative Science and Medicine University of Texas Southwestern Medical Center Dallas Texas USA; ^4^ Cecil H. and Ida Green Center for Reproductive Biology Sciences University of Texas Southwestern Medical Center Dallas Texas USA

## Abstract

Understanding early embryogenesis in livestock remains a persistent bottleneck in both reproductive biology and animal breeding. Work in domestic species has long been limited by restricted access to embryos, ethical considerations, and the complexity of peri‐implantation development, making mechanistic studies difficult. Recent advances in stem cell biology are beginning to shift this. Blastocyst‐like structures, or blastoids, now offer a practical way to model early development in vitro. Blastoids capture key features of natural blastocysts, including overall structure, lineage composition, and gene expression patterns, while being far more accessible experimentally. In livestock species, especially cattle and pigs, blastoid models are starting to gain traction. Livestock blastoids provide a tractable system to study lineage specification, epiblast‐extraembryonic interactions, and early conceptus development. They also open the door to improving assisted reproductive technologies by enabling controlled, scalable experimentation that is not feasible with embryos alone. In this review, we summarize recent advances in the generation of livestock blastoids and discuss how these systems can be used to study early development across livestock species and to inform next‐generation reproductive technologies. We highlight current limitations and outline key challenges ahead, including how blastoid models can be combined with genome editing, stem cell engineering and related embryo‐based approaches to improve reproductive efficiency in domestic animals.

## 1 Introduction

Reproductive efficiency is a key biological trait in livestock production, with direct consequences for herd turnover, genetic gain, cost and long‐term sustainability. In cattle, pigs, sheep, and other domestic species, breeding programmes have progressively incorporated assisted reproductive technologies (ART), including artificial insemination (AI), embryo transfer (ET), cryopreservation, in vitro embryo production (IVP) and somatic cell nuclear transfer (SCNT). More recently, genomic selection and genome editing have further increased the precision and speed of genetic improvement (Hansen 2014; Martinez et al. 2019; Menchaca 2023; Hamze et al. 2025). Despite these advances, early embryonic loss remains a major and unresolved limitation. A substantial proportion of conceptuses fail before implantation or during the period of maternal recognition of pregnancy, particularly in cattle and pigs (Diskin and Morris 2008; Parrilla et al. 2022). This bottleneck is difficult to address because many determinants of developmental competence are established very early, during cleavage, blastocyst formation, lineage segregation, and the initial interactions between the embryo and the maternal environment. While embryo technologies have improved markedly, the molecular and cellular basis of early developmental failure in livestock is still not well defined (Hansen 2014). Experimental access is a major barrier. Early embryos are limited in number, developmentally asynchronous, and sensitive to manipulation. Even in vitro, variability remains high and lineage‐specific perturbations are not straightforward. The peri‐implantation period is even less accessible, particularly in ungulates, where the conceptus undergoes rapid elongation and complex physiological changes that are difficult to recapitulate in culture or follow in vivo. As a result, much of our mechanistic understanding of livestock embryogenesis comes from rodent models. These systems have been indispensable, but they do not fully reflect livestock biology. Domestic species differ from mice in developmental timing, signalling requirements, pluripotent states, lineage allocation, trophoblast behaviour and peri‐implantation morphology. These differences limit the direct translation of findings from rodents to agricultural species.

Stem cell‐based embryo models have emerged as a way to bypass some of these constraints. By exploiting the ability of pluripotent and extraembryonic stem cells to self‐organize, these systems can generate structures that approximate specific stages of early development. Among them, blastoids are particularly relevant because they model the first organized architecture of the blastocyst. In livestock, progress has accelerated in the past few years. Bovine blastoids generated from expanded potential stem cells (EPSCs) together with trophoblast stem cells (TSCs) provided one of the first demonstrations that stem cell–based embryo models can be established in a domestic species (Pinzón‐Arteaga et al. 2023). These structures show morphological and transcriptional features consistent with blastocysts, and they can continue to grow under prolonged in vitro culture conditions. More recently, the derivation and incorporation of bovine extraembryonic endoderm (XEN)‐like stem cells into blastoid assembly improves representation of hypoblast lineages and provides a framework with better representation of embryonic‐extraembryonic crosstalk (Ming et al. 2025). In pigs, chemically induced EPSCs have been used to generate blastocyst‐like structures, extending blastoid approaches to another major livestock species (Liao et al. 2024). Parallel work using porcine embryonic stem cells (ESCs) in defined three‐dimensional differentiation systems suggests that multiple routes to blastoid formation may be feasible, although reproducibility and lineage fidelity still vary across protocols (Xiang et al. 2024). A recent study on sheep blastoids (Cao et al. 2026) reported high‐efficiency blastoid formation by promoting the differentiation of sheep ESCs (sESCs) into trophoblast‐like cells through CDX2‐driven trophoblast induction (iCDX2‐sESC line). The authors demonstrated that iCDX2‐sESCs closely resemble trophoblast cells from preimplantation sheep embryos. Blastoid formation was achieved by co‐culturing iCDX2‐sESCs with sESCs in a 3D system using a two‐step induction protocol. Ovine blastoids formed with high efficiency (~80%) and exhibited morphological, molecular, and lineage characteristics closely resembling those of natural sheep blastocysts. Together, these studies highlight both the promise and the current limitations of livestock blastoids as experimental systems.

In this review, we consider livestock blastoids as emerging models that bridge comparative embryology and reproductive technology. We focus on how these systems can be used to study lineage specification, embryo quality, developmental competence and early conceptus signalling in species of agricultural importance. We also discuss their potential integration with genome editing and stem cell engineering. While still developing, these models offer a path towards more controlled and scalable approaches to studying early development and, ultimately, improving reproductive outcomes in livestock.

## 2 Building Blocks of Livestock Blastoids

The generation of blastoids depends on reconstituting the key cell lineages that first emerge in the blastocyst. In mammals, this begins with segregation of the trophectoderm (TE) and the inner cell mass (ICM), followed by the split of the ICM into epiblast and hypoblast (primitive endoderm). The epiblast forms the embryo proper, while the hypoblast contributes to extraembryonic endoderm tissues, including the yolk sac. In parallel, the TE gives rise to trophoblast lineages that support placental development (Pfeffer 2018; Toyooka 2020). Rebuilding this architecture in vitro therefore requires stem cell populations that can represent, or reliably generate, each of these compartments and interact in a coordinated way.

In livestock species, establishing such stem cell systems has been a long‐standing challenge. Unlike the mouse, where naïve ESCs can be maintained under relatively well‐defined conditions, PSCs from domestic animals have been difficult to derive and stabilize, and often show variable lineage potential (Suasnavas et al. 2015; Bogliotti et al. 2018; Gao et al. 2019; Vilarino et al. 2020). This reflects real biological differences rather than just technical gaps. Livestock embryos differ from rodents in signalling requirements, timing of lineage segregation, and the nature of pluripotency states, all of which complicate efforts to capture equivalent stem cell states in vitro.

That said, the situation has improved in recent years. Multiple stem cell types have now been reported in cattle and pigs, including PSCs with expanded or formative/intermediate features, as well as trophoblast‐like and extraembryonic endoderm‐like cells (Zhang et al. 2019; Soto et al. 2021; Kinoshita et al. 2021; Wang et al. 2025; Su et al. 2026). These advances do not yet match the robustness of mouse systems, but they provide a solid foundation for developing blastoids in livestock species.

### 2.1 Naïve and Primed Pluripotent Stem Cells

Pluripotency in mammals spans a range of cellular states, often simplified as naïve and primed (Du and Wu 2024). Naïve PSCs are typically associated with the preimplantation epiblast and show broad developmental potential, including the capacity to contribute to embryonic lineages in chimera experiments. Primed PSCs, by contrast, resemble a later, post‐implantation epiblast stage and are more lineage‐biassed (Takahashi et al. 2018). While this framework is useful, it is largely defined in mouse and human systems and does not map cleanly onto all species. In livestock, capturing and maintaining a stable naïve‐like state has been difficult. Various culture conditions, often involving inhibition of MEK/ERK and GSK3 pathways with additional modulators, can induce features associated with naïve pluripotency. However, these cells frequently show incomplete or transient naïve characteristics. They tend to be heterogeneous, can drift towards primed‐like states, and often lack robust long‐term self‐renewal (Kumar et al. 2021; Zhi et al. 2022). This instability likely reflects real biological differences rather than technical limitations alone. Livestock embryos differ from rodents in signalling requirements, epigenetic landscape, and the timing of lineage segregation, making it difficult to define a naïve state that is truly equivalent to mouse ESCs. As a result, a consensus ‘ground state’ of naïve pluripotency has not been established for most domestic species. Primed PSCs, in contrast, have been derived more consistently in livestock. These cells are typically established from blastocysts or early embryonic tissues and maintained under conditions that include activation of FGF and Activin/Nodal signalling, as well as inhibition of canonical WNT pathway, which support a post‐implantation‐like identity (Choi et al. 2019; Soto et al. 2023). Although primed cells lack the broad developmental potential attributed to naïve states, they are generally more stable in culture, which has made them the most widely used pluripotent platform in these species (Choi et al. 2024; Shirasawa et al. 2024).

### 2.2 Expanded Potential Stem Cells

Expanded potential stem cells (EPSCs) have been proposed as an alternative pluripotent state with broader developmental capacity than conventional naïve or primed cells (Yang, Ryan, et al. 2017; Yang, Liu, et al. 2017). They were initially defined by reports of contribution to both embryonic and extraembryonic lineages, including trophectoderm and primitive endoderm, suggesting features of earlier preimplantation stages. This idea has been particularly appealing in livestock, where stable naïve pluripotent states have been difficult to establish. EPSCs are typically derived and maintained by modulating multiple signalling pathways, including WNT and MAPK/ERK, along with pathways linked to cell polarity and Hippo signalling. Under these conditions, cells often show transcriptional signatures associated with early embryos and display greater developmental flexibility than standard PSCs. EPSC‐like lines have now been reported in livestock species such as cattle and pigs, where they can be propagated in vitro and used to generate both embryonic and extraembryonic cell types (Gao et al. 2019; Zhao et al. 2021; Xiang et al. 2021). In practice, this expanded potential has made EPSCs useful for building blastoids, since a single starting population can be directed into multiple lineages. This is an advantage in livestock, where fully defined trophoblast and hypoblast stem cell lines are still limited.

Despite the potential, however, the biological identity of EPSCs remains unsettled. It is unclear whether they correspond to a discrete in vivo cell state or represent an induced condition with broadened, but artificial, potential. Reported contributions to extraembryonic lineages, especially trophectoderm, have been inconsistent and often depend on the experimental context. EPSC cultures are also sensitive to derivation and maintenance conditions and can be heterogeneous.

Overall, EPSCs are best viewed as a practical workaround rather than a resolved pluripotent state. They bridge, to some extent, the gap between pluripotent and extraembryonic systems and have enabled progress in in vitro embryo modelling in livestock. However, their developmental equivalence to early embryonic stages in vivo remains to be defined more rigorously.

### 2.3 Extraembryonic Stem Cells

In addition to PSCs, the derivation of extraembryonic stem cell types has been an important step towards building blastocyst‐like systems in livestock. These include trophoblast stem cells (TSCs) and extraembryonic endoderm (XEN) cells, which correspond broadly to the trophoblast and hypoblast lineages. Compared to PSCs, extraembryonic lineages have in some cases been easier to capture in vitro, although stable and well‐defined systems are still limited.

TSCs represent the epithelial lineage that forms the placenta and are central to early conceptus development, particularly in ungulates where trophoblast proliferation drives conceptus elongation and maternal recognition of pregnancy. In cattle, several studies have reported the derivation of TSC‐like lines that can be expanded in vitro and express core trophoblast markers, with evidence of epithelial organization and the ability to differentiate along trophoblast trajectories (e.g., binucleate‐like or elongated trophoblast states) (Wang et al. 2021, 2023; Baquerre et al. 2025; Abdelhady et al. 2026). These systems provide a useful entry point to study bovine trophoblast biology, which has historically been difficult to access experimentally. That said, their equivalence to in vivo TE and later trophoblast subtypes is not fully resolved. Culture conditions remain variable across studies, and differentiation capacity is still limited compared to what is observed in the conceptus. In pigs, trophoblast‐like cells have been generated from embryos and through directed differentiation of PSCs (Hou et al. 2015; Kim et al. 2024). However, the establishment of stable, long‐term self‐renewing TSC lines with clear and consistent identity has been less successful. This likely reflects species‐specific differences in trophoblast biology and signalling requirements, and highlights the need for tailored culture systems rather than direct transfer of mouse or human conditions.

The hypoblast lineage has been even more difficult to capture in livestock. The recent derivation of bovine XEN‐like cells therefore marks a notable advance. These cells can be established from blastocysts under defined conditions and show transcriptional features consistent with primitive endoderm, including expression of markers such as SOX17 and GATA6 (Ming et al. 2025). They can be maintained over multiple passages and, in co‐culture settings, appear to support interactions with pluripotent cells, suggesting some degree of functional relevance. Still, these systems are at an early stage. Their correspondence to in vivo hypoblast, particularly during later peri‐implantation stages, remains to be defined. In pigs and other livestock species, XEN‐like stem cell lines have not been as clearly established. Hypoblast‐like populations are typically generated through differentiation of PSCs rather than maintained as independent stem cell types (Park et al. 2021). Overall, while progress in extraembryonic stem cell derivation has been significant, especially in cattle, these systems are not yet as robust or standardized as their mouse counterparts. Further work will be needed to define their developmental equivalence and to integrate them into more complete embryo models.

## 3 Livestock Blastoids: From Proof of Concept to Emerging Embryo Models

Blastoid systems have now been reported in cattle, pigs and sheep, using approaches that reflect differences in available stem cell types and how lineage specification is handled in each species (Ming et al. 2026). In cattle and sheep, most strategies have relied on combining multiple cell populations, including pluripotent or EPSCs with trophoblast and, more recently, extraembryonic endoderm‐like cells, to approximate blastocyst organization. In pigs, blastoid formation has more often been achieved through directed differentiation or self‐organization of pluripotent or EPSCs under defined culture conditions. These approaches are summarized in Figure 1. While these systems capture aspects of blastocyst structure and lineage segregation, they are still best viewed as early‐stage models rather than fully functional equivalents of embryos.

**FIGURE 1:**
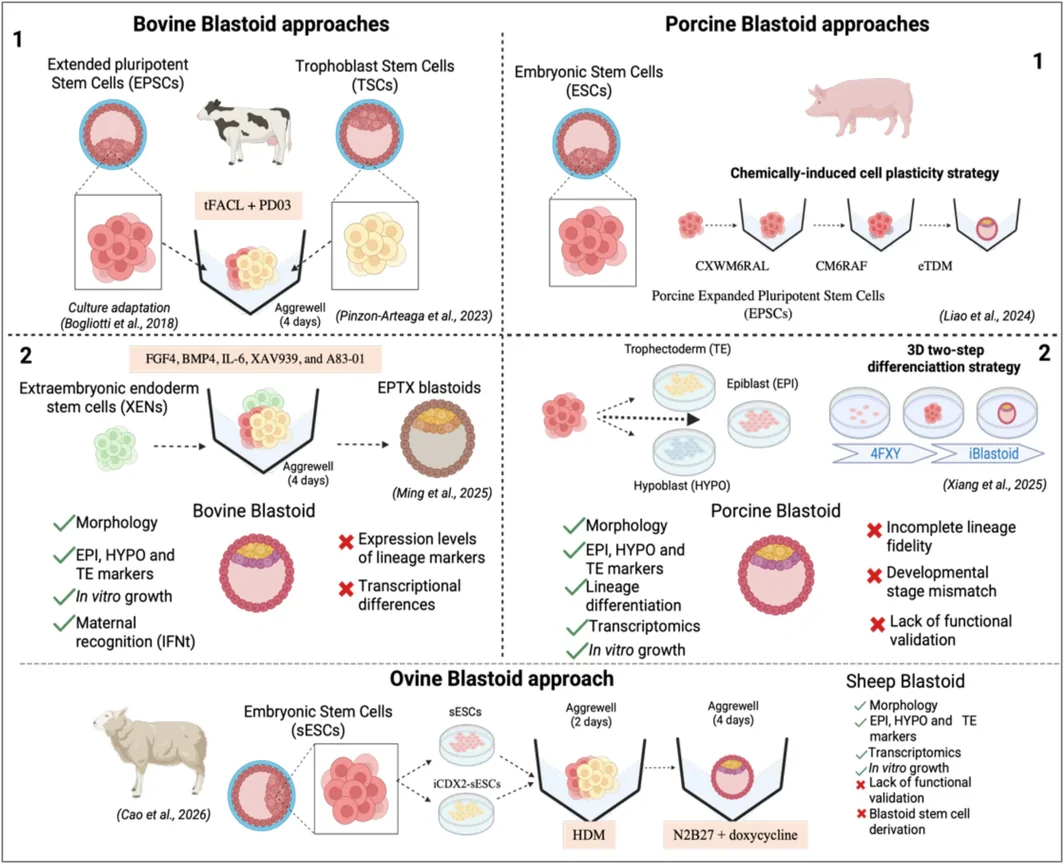
Stem cell‐based strategies for blastoid generation in livestock species. Bovine blastoids are generated through the assembly of expanded potential stem cells (EPSCs) and trophoblast stem cells (TSCs), with further refinement achieved by the incorporation of extraembryonic endoderm stem cells (XEN). In contrast, porcine blastoids are derived from expanded pluripotent stem cells (EPSCs) or embryonic stem cells (ESCs) via Chemically inducing cell plasticity or using a two‐step three‐dimensional differentiation strategy under defined culture conditions. Ovine blastoids are generated through the co‐incubation of ESCs and trophoblast‐like cells (TLSCs).

### 3.1 Bovine Blastoids

The first demonstration of stem cell‐based embryo model in a livestock species was established in cattle. In that study, bovine EPSCs were combined with TSCs in a three‐dimensional culture system that supports lineage organization and cavitation (Pinzón‐Arteaga et al. 2023). The resulting structures formed with relatively high efficiency and showed several features associated with in vivo blastocysts. Morphologically, bovine blastoids developed a blastocoel‐like cavity, an outer epithelial layer resembling trophectoderm, and an inner cell mass‐like compartment, broadly similar to day 7–8 bovine blastocysts. Immunostaining detected cells expressing lineage‐associated markers, including SOX2 (epiblast), SOX17 (hypoblast), and trophoblast markers such as GATA3, KRT18 and CDX2. Epithelial polarity and junctional organization were also evident, consistent with basic blastocyst architecture. At the transcriptomic level, single‐cell analyses placed blastoid cells along trajectories corresponding to embryonic and extraembryonic lineages, supporting the idea that these systems can approximate early lineage segregation. At the same time, differences from embryos are clear. Lineage proportions are not well matched, with hypoblast‐like cells often underrepresented and epiblast‐like cells relatively enriched. Marker expression also differs in magnitude and consistency across lineages, and the overall transcriptional state does not fully align with in vivo counterparts. These gaps point to incomplete lineage specification and suggest that current systems capture only part of the underlying biology. One useful feature of the bovine system is that both blastocysts and blastoids can be maintained in extended three‐dimensional culture, where they continue to expand and show trophoblast proliferation. This begins to provide experimental access to stages that bridge pre‐implantation and early conceptus development, which are otherwise difficult to study in cattle. There is also preliminary evidence that, after transfer, blastoid‐derived structures can elicit aspects of maternal recognition of pregnancy (Pinzón‐Arteaga et al. 2023). However, whether bovine blastoids can undergo sustained peri‐implantation development and conceptus elongation comparable to in vivo embryos remains unclear.

Subsequent work has focused on improving lineage representation. The addition of bovine XENs to the assembly generates more complex EPTX blastoids, with improved hypoblast representation and closer alignment to blastocyst lineage composition and gene expression (Ming et al. 2025). This supports a broader point: the hypoblast is not just a passive lineage but likely plays an active role in coordinating blastocyst organization and developmental progression.

Overall, bovine blastoids have moved beyond proof of concept and now provide a workable, if still imperfect, model of early development. The main challenge is no longer whether these structures can be generated, but how to improve their fidelity and define the specific questions they can answer better than embryos or existing in vitro systems.

### 3.2 Porcine Blastoids

The pig provides a useful and complementary system for blastoid research. Beyond its importance as a livestock species, it is widely used in biomedical studies because of its physiological and anatomical similarities to humans. This makes porcine models relevant not only for agriculture but also for translational work. In contrast to bovine systems, where blastoid formation has largely relied on combining multiple lineage‐restricted cell types, most porcine approaches have focused on the self‐organization of a single pluripotent or EPSC population, similar to the generation of human blastoids (Yu, Wei, Duan, et al. 2021, Yu, Wei, Sun, et al. 2021, Yu et al. 2023). One strategy uses chemically induced EPSCs that show features associated with early embryonic states and can generate blastocyst‐like structures under three‐dimensional culture conditions (Liao et al. 2024). These structures display basic blastocyst architecture, including cavity formation and segregation of embryonic and extraembryonic‐like compartments, although the degree of fidelity varies across studies. A parallel approach has been developed using porcine ESCs maintained under defined culture conditions (4FIXY medium), a system combining 4 cytokines and small‐molecule inhibitors that support a stable primed pluripotent state (Xiang et al. 2024). When subjected to three‐dimensional differentiation, these cells can form blastocyst‐like structures that resemble porcine blastocysts in size, morphology and aspects of lineage marker expression. Single‐cell transcriptomic analyses place these cells along trajectories consistent with epiblast and extraembryonic lineages, although, as in other systems, the correspondence to in vivo embryos is incomplete.

Similar to bovine, porcine blastoids can also be maintained and expanded in vitro for extended periods, providing a platform to study early developmental events that are otherwise difficult to access. At the same time, lineage specification is less tightly controlled than in multi‐lineage assembly systems, and hypoblast and trophoblast compartments are often less well defined or require directed induction rather than emerging robustly.

Overall, porcine blastoids highlight a different route to generate blastocyst‐like structures, where self‐organization from a single stem cell population is used. This suggests that blastoid formation does not depend on a single strategy but can be achieved through distinct starting cellular states and differentiation paths.

### 3.3 Ovine Blastoids

A recent study has established a robust strategy for generating ovine blastoids (Cao et al. 2026). Through transcriptomic analyses of preimplantation sheep embryos, the authors identified CDX2 as a key regulator of ovine trophoblast lineage. Overexpression of CDX2 in sESCs (iCDX2‐sESCs) resulted in cells showing molecular and functional features consistent with trophoblast cells, including the upregulation of canonical trophoblast markers and the ability to differentiate into specialized trophoblast subtypes. Building on this, ovine blastoids were generated by co‐culturing iCDX2‐sESCs with sESCs in a 3D aggregation system. The authors implemented a two‐step induction strategy designed to better mimic peri‐implantation dynamics. In the first phase, small aggregates (~25 cells) were cultured in hypoblast differentiation medium (HDM) supplemented with FGF2, activin A and CHIR99021 to promote the emergence of hypoblast‐like cells (HLCs). After 2 days, once compact aggregates had formed, the culture was transitioned to N2B27 medium containing doxycycline to maintain CDX2 induction, together with a low dose of chemical inhibitors to stabilize lineage segregation and promote further development. This optimized condition, referred to as N2B27‐aggregate medium (HDM‐AM‐#2), markedly improved blastoid formation efficiency, reaching approximately 80%. The generated structures closely resembled natural blastocysts in both organization and composition, displaying an ICM‐like compartment and a surrounding trophectoderm‐like layer enclosing a central cavity. Their size and morphology were comparable to ovine blastocysts, and they contained the three main lineages: epiblast‐, hypoblast‐ and trophectoderm‐like cells, in proportions similar to those observed in ovine blastocysts. Transcriptomic analyses further confirmed a strong similarity to natural embryos, with lineage‐specific gene expression patterns consistent with their expected developmental roles. Functionally, these blastoids showed partial developmental potential, as they could give rise to embryonic and extraembryonic endoderm stem cell lines and were able to survive and expand in vitro for extended periods, although with reduced long‐term viability compared to natural blastocysts.

## 4 Blastoids as Platforms for Reproductive Technologies in Livestock

The value of livestock blastoids lies in their potential to connect developmental biology with practical problems in reproduction. The key question now is what they can do better than existing systems. One immediate application is in optimizing embryo culture conditions. In vitro embryo production in livestock is still limited by variability in developmental competence and an incomplete understanding of how culture conditions shape early lineage decisions. Access to in vivo embryos is limited and costly, which makes systematic testing difficult. Blastoids offer a more scalable and standardized system. They can be used to compare media composition, oxygen tension, growth factors, extracellular matrices, and timing of culture changes, while monitoring effects on lineage allocation, cavity formation, epithelial integrity and gene expression. These readouts overlap with features linked to embryo quality, making blastoids a useful screening tool, although findings will still need to be validated in embryos.

A second area is functional genetics. Because blastoids are derived from stem cells, they are well suited to genome editing. Genetic perturbations can be introduced in starting pluripotent and extraembryonic stem cells, followed by assessment of effects on lineage specification, structural organization and early developmental behaviours. This is particularly useful in livestock, where direct genetic testing in embryos is technically demanding and low‐throughput. Blastoid systems could be used to prioritize candidate genes involved in early development, including those linked to embryo survival, trophoblast function and early conceptus signalling, before moving to in vivo validation.

A third application is the study of early embryonic loss. A large fraction of conceptuses in livestock fail before or around implantation, often without a clear cause. Blastoids are not fully functional embryos, but they allow specific processes to be isolated and tested under controlled conditions. These include lineage imbalance, defects in trophoblast epithelialization, disrupted embryonic‐extraembryonic interactions, and responses to environmental stress. In this sense, blastoids can shift the focus from descriptive outcomes to experimentally testable mechanisms.

A fourth area is conceptus signalling and embryo–maternal interactions. There is preliminary evidence that bovine blastoid‐derived structures can produce signals associated with maternal recognition of pregnancy after transfer, although these observations are still limited. Even without full developmental competence, trophoblast‐rich blastoid systems may capture aspects of early conceptus signalling. This creates an opportunity to study the initial molecular dialogue between the conceptus and the uterus, which is central to pregnancy establishment in species such as cattle and pigs.

Overall, blastoids are unlikely to replace embryos, but they provide a tractable system for questions that are otherwise difficult to approach. Their main value will depend on how well they can be integrated with existing embryo‐based and in vivo models.

## 5 Current Limitations

The excitement around livestock blastoids should be balanced with a clear view of their limitations. The main issue is fidelity. Similarity to a blastocyst in shape and marker expression is a starting point, but it is not enough. A useful model needs to be judged across multiple features, including lineage proportions, spatial organization, transcriptional and epigenetic profiles, developmental timing and functional outputs. By these measures, current livestock blastoids remain incomplete. A second limitation is that different generation strategies introduce different biases. Assembly‐based approaches can reproduce overall structure more consistently, but they depend heavily on the quality and definition of the input cell types. Self‐organizing systems derived from pluripotent or EPSCs are more flexible, but lineage specification is often less controlled and more variable. As a result, differences between bovine and porcine blastoids may reflect both biology and methodology, and the two are not always easy to separate. A third constraint is stage coverage. Most current systems capture aspects of blastocyst formation and early expansion, but they do not yet reproduce the full progression of peri‐implantation development. This gap is especially important in livestock, where events after the blastocyst stage, such as conceptus elongation and species‐specific signalling, play a central role in pregnancy establishment. A fourth issue is standardization. Another major unresolved issue is whether livestock blastoids can support sustained peri‐implantation development after transfer, including conceptus elongation in ungulates. Although preliminary studies suggest that bovine blastoids may transiently induce aspects of maternal recognition of pregnancy, elongation and full developmental progression have not yet been demonstrated. The field is still developing, and there is no widely accepted framework for defining what constitutes a livestock blastoid, how closely it should match an embryo, or which assays are most informative for functional relevance. Without clearer benchmarks, it remains difficult to compare results across studies or to assess how improvements in one system translate to another.

## 6 Future Directions: Towards Functional, Comparative and Translational Livestock Embryo Models

The next phase of livestock blastoid work will depend less on generating structures and more on defining what they can do reliably. Several directions stand out. One is improving lineage completeness and definition. Progress will likely depend on better characterization of stem cell states that correspond to epiblast, trophoblast and hypoblast lineages in each species. At present, many of these states are defined operationally rather than functionally. More precise mapping to in vivo counterparts, along with improved culture systems, should lead to blastoids with more balanced lineage composition and more consistent embryonic–extraembryonic interactions. A second direction is comparative analysis. With blastoid systems now available in cattle, pigs and sheep, there is an opportunity to examine how early lineage specification, trophoblast behaviour and pluripotency states differ across species under controlled conditions. This is not just a technical comparison. Livestock species diverge in key aspects of early development, including timing of lineage segregation and peri‐implantation morphogenesis. Blastoids could provide a practical way to study these differences side by side. A third direction is integration with reproductive technologies. Blastoid‐based assays could be incorporated into workflows for testing culture conditions, benchmarking stem cell lines, or prioritizing candidate genes involved in early development. They may also be useful for assessing trophoblast function and early conceptus signalling in vitro. These applications will depend on how well blastoid readouts correlate with outcomes in embryos, which remains to be established. More broadly, livestock blastoids may help refine how embryo quality is defined. In practice, embryo quality is still judged largely by morphology or survival after transfer. Blastoid systems offer a way to probe underlying features such as lineage balance, structural organization and signalling capacity. Whether these measures translate into improved reproductive outcomes is an open question, but they provide a more mechanistic framework than current criteria. Another important future direction will be extending blastoid systems to additional livestock species, including goats and horses (Yu, Wei, Duan, et al. 2021, Yu, Wei, Sun, et al. 2021), where species‐specific differences in pluripotency, trophoblast development and peri‐implantation biology may reveal new principles of early embryogenesis and reproductive adaptation. Overall, the value of livestock blastoids will depend on moving from descriptive resemblance to measurable function. That shift, rather than further increases in efficiency or yield, is likely to determine their long‐term impact.

## 7 Conclusions

Livestock blastoids are emerging as a new class of in vitro models with relevance for both developmental biology and animal reproduction. In cattle, pigs and sheep, recent work shows that stem cells can be directed to form blastocyst‐like structures that capture key features of early embryos, including basic architecture, lineage segregation and aspects of early growth in culture. Their value lies less in resemblance and more in use. Blastoids provide a tractable system to study lineage specification, embryonic‐extraembryonic interactions and early conceptus signalling under controlled conditions. They also offer a scalable platform to test culture conditions, probe gene function and explore mechanisms underlying embryo competence and early pregnancy loss in livestock. At the same time, these systems are still incomplete. They do not fully match embryos in lineage balance, developmental progression, or functional capacity. Rather than replacing embryos, they should be viewed as complementary tools that make early developmental processes easier to access and manipulate. If improved with better‐defined cell states and validated against in vivo outcomes, livestock blastoids could become a practical bridge between mechanistic studies of early development and efforts to improve reproductive efficiency in domestic species.

## Author Contributions

Cristina A. Martinez‐Serrano and Jun Wu conceived and wrote the manuscript. Cristina A. Martinez‐Serrano prepared the figure with input from Jun Wu.

## Conflicts of Interest

The authors declare no conflicts of interest.

## Data Availability

Data sharing not applicable to this article as no datasets were generated or analysed during the current study.
